# The synergism of lytic polysaccharide monooxygenases with lichenase and their co-immobilization on silica nanospheres for green conversion of lichen biomass

**DOI:** 10.3389/fnut.2022.970540

**Published:** 2022-10-19

**Authors:** Lixi Cai, Ying Zheng, Yunmeng Chu, Yuanqing Lin, Lixing Liu, Guangya Zhang

**Affiliations:** ^1^College of Basic Medicine, Putian University, Putian, China; ^2^Department of Bioengineering and Biotechnology, Huaqiao University, Xiamen, China; ^3^Key Laboratory of Translational Tumor Medicine in Fujian Province, Putian University, Putian, China; ^4^College of Pharmaceutical and Medical Technology, Putian University, Putian, China

**Keywords:** lytic polysaccharide monooxygenase, synergistic effect, lichenase, silica nanoparticles, multi-enzyme immobilization

## Abstract

Enzyme-assisted valorization of lichenan represents a green and sustainable alternative to the conventional chemical industry. The recently discovered lytic polysaccharide monooxygenases (LPMOs) are essential components of state-of-the-art enzyme cocktails for lichenin bioconversion. The LPMOs named SpyTag fused LPMOs (AST) from *Chaetomium globosum* was functionally expressed in *E. coli* and exhibited 1.25-fold synergism with lichenase, whereas AST alone produced no detectable reducing sugars. HPLC results further confirm that AST does not alter the endogenous hydrolysis mode of lichenase but rather enhances its hydrolysis efficiency by disrupting the long chain of lichenan and releasing more reducing ends. To the best of our knowledge, this was the first report on the synergistic effect of LPMOs and lichenase, which may have great synergistic potential in the conversion of lichen biomass. Furthermore, a novel strategy for the covalently immobilizing AST and lichenase on silica nanoparticles (SNPs) from the cell lysate in a single step was proposed, which exhibited high activity recovery (82.9%) and high immobilization yield (94.8%). After 12 independent runs, about 67.4 % of the initial activity of the immobilized enzymes was retained. The resulted biocatalyst systems exhibited the green and sustainable strategy in the bioconversion of lichen biomass as well as other diverse polysaccharides.

## Introduction

Lichens, the symbionts of fungi and algae, have attracted wide attention due to their abundant sources, huge reserves, and low production costs. Lichenan (β-1,3-1,4-glucan) is a linear polysaccharide derived from lichen, accounting for approximately 62% of the total carbohydrates in lichen, but the high viscosity of lichens in water results in low utilization (1). Lichenase (EC 3.2.1.73) could cleave linear lichenan consisting of β-1,3 and β-1,4 bonds with strict specificity for the β-1,4 glycosidic bond adjacent to the 3-O-glucopyranose residue (2–4), which can reduce viscosity for processing into biofuels (i.e., ethanol) or high value-added products (5, 6). Lichenase plays a promising role in the green conversion of lichen biomass (7, 8). However, lichenase suffers from some drawbacks that may impede its industrial application, such as time-consuming purification processes, thermal instability, and poor reusability. Thus, combining existing lichenase with co-proteins to reduce the enzyme load or adopting enzyme immobilization technology may effectively solve these deficiencies (9).

Lytic polysaccharide monooxygenases (LPMOs) are novel copper ion-dependent biomass-degrading enzymes (10), with copper ions bonded to the characteristic histidine scaffold and endowing LPMOs with a significant oxidative capacity (11–13). LPMOs can oxidatively cleave glycosidic bonds in polysaccharides that cannot be inaccessibly cleaved by glycosidic hydrolases, such as cellobiohydrolases or endoglucanases. By increasing the accessibility of substrates and generating more reducing ends, LPMOs enhance the overall efficiency of glycosidases in degrading insoluble polysaccharides (14). Thus, LPMOs are novel biomass-degrading enzyme that enriches and changes the paradigm of biomass conversion (15, 16). Dimarogona found that in the presence of a reductant, the loading of LPMOs from *Sporotrichum Thermophile* resulted in a 20% increase in oligosaccharide recovery from pretreated spruce (16). Meanwhile, by loading LPMOs to Novozymes’ cellulase system in Denmark, the production cost of cellulosic ethanol has approached that of starch ethanol or gasoline, down to about $2/gallon. LPMOs have been found to significantly boost the efficiency of the enzymatic digestion of cellulase, 1,4-xylanase, amylase, and chitosanase, making them an ideal synergistic enzyme for the degradation of polysaccharides (17–19). However, there are no studies on the synergistic effect of lichenase by LPMOs. Besides, the studies on the synergistic degradation of biomass by LPMOs are all based on free enzymes. Therefore, exploring more glycoside hydrolases that can synergize with LPMOs and establishing a reliable multi-enzyme immobilization strategy for large-scale use of LPMOs in biorefineries may become an essential field for biomass processing in the future.

In recent years, the idea of biomimetic mineralization has been gradually introduced into the design and preparation of immobilized carriers (20). Among them, silica carriers obtained by biomimetic silicification have attracted much attention due to their mild and efficient preparation conditions, well-ordered structure, and high thermal stability (21, 22). Recently, our team found that cationic elastin-like polypeptides (ELPs) have the ability to rapidly prepare biomimetic silica nanoparticles (SNPs). ELPs are temperature-responsive and can be obtained with high purity by the simple multiple reversible phase transition cycling (ITC) method (23, 24). Thus, ELPs are endowed with dual functions of biomimetic silicification and purification tags, and allow biomimetic mineralization to prepare inexpensive carriers that simplify the immobilization process. However, two or more enzymes are often required for synergistic hydrolysis in the hydrolysis of lichenan. Multi-enzyme immobilization can thoroughly combine the catalytic properties of different enzymes and improve the overall reaction efficiency. The enzymes in traditional immobilization methods are non-specifically bound to the carrier with multiple sites, resulting in different degrees of enzyme activity. Fusion expression, separation, and purification of multi-enzymes and molecular adhesives, and then immobilization on biomimetic siliconized carriers is an effective way to solve this problem. It can also shorten the distance between enzymes, generate substrate channels, and improve the overall catalysis performance.

Herein, the LPMOs derived from the fungus *Chaetomium globosum* was successfully expressed in *E. coli* and explored its synergistic effects with lichenase, that is, the first report on the synergistic effect of lichenase and LPMOs to our best knowledge. Then we introduced lysine into acidic ELPs to design basic ELPs with biomimetic ability (K5V4F) and then fused them with the molecular binder SpyCatcher (K5SC). The temperature-responsive of ELPs was used to isolate, and purify the fusion proteins and combine the biomimetic ability of ELPs to generate silica nanocarriers (K5SC@SiO_2_). Meanwhile, SpyTag fused lichenase (BST) and SpyTag fused LPMOs (AST) were designed and synthesized, since SpyTag rapidly forms a spontaneous intermolecular isopeptide bond to its partner SpyCatcher (25, 26). As a result, K5SC@SiO_2_ can intelligently capture the target dual-enzyme (BST and AST) containing SpyTag, enabling self-immobilization of the dual-enzyme. This green strategy allows for the purification and self-immobilization of multi-enzyme from cell lysates in one step, which may have great potential in the bioconversion of lichen biomass as well as other diverse polysaccharides.

## Materials and methods

### Plasmid construction and protein expression

AST and BST: the genes encoding for molecular binder SpyTag were synthesized by Genscript and then fused to the N-terminus of the LPMOs (No. MN190001) and lichenase (BglS, No. 937470) respectively, resulting in pET-22bST-A (AST), and pET-22bST-B (BST) respectively. BglsE: the genes encoding for lichenase (BglS, No. 937470) were synthesized by Sangon Biotech and then ligated into the pET-22b-ELPs [KV8F-40] at the N-terminus of the ELPs, resulting in pET-22bBglS-ELP (BglsE). K5SC: the genes encoding for molecular binder SpyCatcher were synthesized by Sangon Biotech and then ligated into the pET-22b-ELPs [K5V4F-40] at the N-terminus of the ELPs, resulting in pET-22bSC-K5 (K5SC, No. MN136291).

The resulting plasmids were transformed into BL21 (DE3) cells for expression in *E. coli*, and incubated in a fresh TB medium containing 100 mg/mL ampicillin for 4 h at 37°C to make the OD600 reached 0.4, and then loaded with 0.5 mmol/L IPTG. Cells were collected by centrifugation at 4,000 × *g* for 20 min and sonicated on ice. The sonicated cell suspension was centrifuged at 12,000 × *g* for 20 min to remove insoluble cell debris, and the supernatant contains cell lysates of AST, BST, and K5SC. Then the cell lysate of AST was purified by using a Ni-affinity column (Smart-Lifesciences, China) with elution buffer (50 mmol/L Tris–HCl buffer, 80 mmol/L imidazole, and 500 mmol/L NaCl, respectively).

The recombinant protein of K5SC and BglsE were purified by the modified ITC cycle. Briefly, a final concentration of 2.5 mol/L NaCl was loaded to the cell lysate to trigger the phase transition aggregation of K5SC at 37°C (27). The resulting aggregates were collected from the cell lysate by simple centrifugation at 4°C (13,400 × *g*, 10 min). Subsequently, the aggregates of K5SC and BglsE were resuspended in ice PBS buffer for 60 min and centrifuged at 4°C (13,400 × *g*, 20 min) to remove the impurities. The ITC process was repeated twice, as mentioned above, to obtain high-purity recombinant protein of K5SC and BglsE.

### Synergistic effects of lytic polysaccharide monooxygenases on lichenase

Different molar ratios of AST and BglsE (0:1, 1.6:1, 2.6:1, 3.2:1, 4:1, 6.4:1, 16:1, and 32:1; the amount of BglsE was fixed at 2.4 mg/g) were added to citrate phosphate buffer (CPB, 20 mmol/L, pH 6.6). To terminate the enzymatic reaction, 3,5-dinitrosalicylic acid (DNS) was added to the reaction mixture and heated at 100°C for 5 min, then centrifugated at 12,000 × *g* for 8 min to remove the precipitate. Temperature (25–60°C) and pH (4.0–8.0) changes were measured at 540 nm using D-glucose as the reducing sugar standard. The time profile was measured by 2.4 mg/g lichenase along with 4.64 mg/g AST at 50°C for 36 h to measure the reducing sugar yield at each time period (Kim et al. (28). The degree of synergy (DS) was determined as follows.

$$\text{Ds}=\frac{M_{A\,B}}{M_{B}}$$

Where M_AB_ was the yield of reducing sugars produced by AST and BglsE, and M_B_ was the yield of reducing sugars produced by BglsE alone.

The relative enzyme activities were calculated by measuring the activities of BglsE and AST before and after immobilization with EDTA and metal cations (Mn^2+^, Zn^2+^, K^+^, Mg^2+^, Cu^2+^, Ba^2+^, Ni^2+^, Co^2+^, Ca^2+^) at a final concentration of 10 mmol/L respectively.

### High-performance liquid chromatography

To characterize the composition of enzymatic digestion products from lichenan *via* the synergistic effect of lichenase and LPMOs, the high-performance liquid chromatography (HPLC, Agilent 1260) equipped with an oscillometric refractive index detector and Waters Sugar-Par™ column (USA, 5 μm, 6.5 mm × 300 mm) were used. Elute the sample solution with ultrapure water at a flow rate of 0.5 mL/min at a column temperature of 75°C and a detector temperature of 40°C (29).

### Biomimetic silicification and carrier characterization

The SiO_2_ carriers were synthesized by biomimetic mineralization using K5SC as the catalyst and TMOS as the silica source. Briefly, K5SC protein (300 μmol/L) and TMOS (1 mol/L) were mixed thoroughly in a volume ratio of 9:1 at room temperature for 5 min. The auto-formed K5SC@SiO_2_ carriers were separated from the reaction system by centrifugation at 5,000 × *g* for 3 min. The size and surface morphology of K5SC@SiO_2_ were studied by FESEM.

### Purification and immobilization of lichenase and lytic polysaccharide monooxygenases

The recombinant enzyme of AST and BST were covalently purified and immobilized onto the K5SC@SiO_2_ NPs by the modified SpyCather/SpyTag reaction (30). Briefly, the above-prepared K5SC@SiO_2_ were loaded into the cell lysate AST and BST, mixed thoroughly, and reacted for 1 h at 30°C in a constant temperature vortex mixer. After centrifugation at 5,000 × *g* for 3 min at 4°C, the precipitate was collected and suspended in an equal volume of Tris–HCl buffer (pH 7.0) to form the immobilized enzyme solution. To make the electrophoresis results after immobilization clearer, we added some pure AST into the cell lysate for K5-C@SiO_2_ capture. However, pure AST was not added in other experiments. The method described by Sheldon uses three indicators were used to analyze the immobilization effect of enzymes: immobilization yield, immobilization efficiency, and activity recovery (31).

$$\mathrm{Immobilizationyield}\left(\%\right)=\frac{\mathrm{At}}{\mathrm{Ao}}\times 100\%$$

$$\mathrm{Activityrecovery}\left(\%\right)=\frac{\mathrm{As}}{\mathrm{Ao}}\times 100\%$$

$$\mathrm{Immobilizationefficiency}\left(\%\right)=\frac{\mathrm{As}}{\mathrm{At}}\times 100\%$$

where:

Ao: Starting activity of free enzyme; As: Observed activity;

At: Immobilized activity.

### Reusability of the immobilized enzyme

The reusability of immobilized AST and BST were measured at optimum conditions for 10 min, the immobilized biocatalyst was immediately centrifuged at 4°C (1 min 12,000 rpm), and the supernatant was collected for determination of enzyme activity at each cycle. The recycled precipitate was washed with buffer to remove residual sugar before adding the new lichenan substrate for the next cycle of experiments. The activity of the first cycle was defined as 100%, and a total of 12 rounds were repeated.

## Results and discussion

### Expression and purification of the recombinant proteins

The purified proteins of BglsE and AST were verified by SDS-PAGE with the clear band around each of the molecular weights at 39 kDa (Supplementary Figure 1A, lane 1) and 25 kDa (Supplementary Figure 1B, lane 1), which were in general agreement with their theoretical values. The final yields of BglsE and AST were 7.46 ± 0.39 mg and 0.68 ± 0.08 mg per 200 mL of fermentation culture tested by the BCA kit. The preparation process of BglsE protein only requires simple centrifugation technology and cheap reagents such as NaCl, indicating that the expensive and cumbersome chromatographic purification process can be avoided, and the large-scale production of lichenase can be easily prepared with only two rounds of ITC.

### Synergistic or stabilizing effect of lytic polysaccharide monooxygenases

**FIGURE 1:**
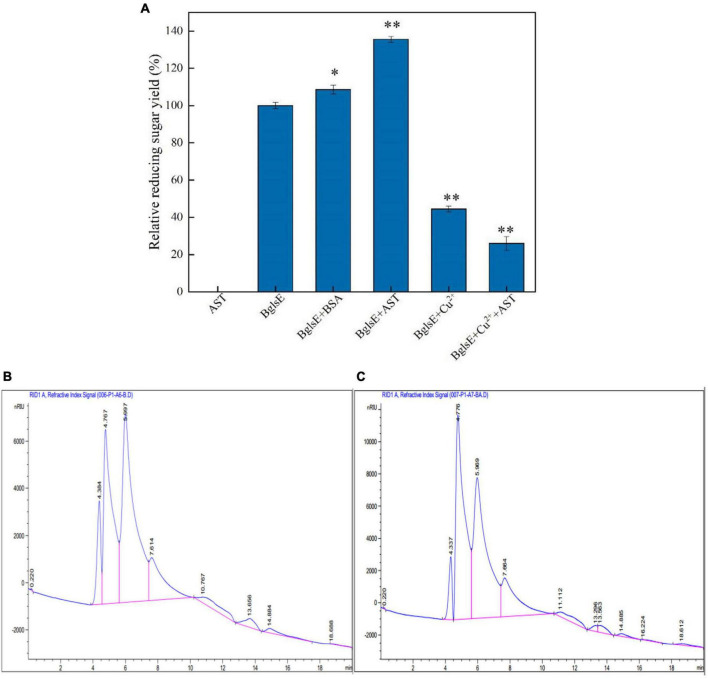
**(A)** Effects of LPMOs, Cu^2+^, and BSA on lichenase activity. **(B)** Product profiles of the hydrolysis reactions of BglsE analyzing by HPLC. **(C)** Product profiles of the hydrolysis reactions of BglsE and AST analyzing by HPLC. *Significant difference from the control (*p* < 0.05); ^**^very significant difference from the control (*p* < 0.01).

When purified AST was incubated with insoluble lichenan for 2 h, no detectable yield of reducing sugars could be detected (Figure 1A). However, the yield of reducing sugars produced by co-incubation of BglsE and AST for 2 h was significantly higher than that of BglsE alone, with a synergistic effect of 1.25-fold, indicating that AST does not directly hydrolyze lichenan but enhances the hydrolytic efficiency of lichenase. AST, a typical metalloprotease is often activated by the reduction of Cu^2+^ in coordination with the catalytic center of the LPMOs (32, 33). To exclude the effect of Cu^2+^ on the enzymatic activity of lichenase, 10 mmol/L Cu^2+^ were loaded in the BglsE alone or BglsE/AST, which found that Cu^2+^ had a strong inhibitory effect on the activity of both BglsE (44.48%) or BglsE/AST (25.99%). Furthermore, to verify whether the effect of AST on BglsE was based on stabilizing or synergistic effect, bovine serum albumin (BSA) was chosen as the additional control protein because it is widely applied in the stabilization of enzymes (28). Comparing the effects of AST and BSA on the hydrolysis rate of lichenan, the improvement of AST on the relative reducing sugar yield was significantly greater (e.g., 1.25-fold, *p* < 0.01) than that of BSA with the same molar mass (e.g., 1.08-fold, *p* < 0.05), indicating that AST can act on C1, C4, or C1, C6 on lichenan, and it has a synergistic rather than a stabilizing effect with lichenase. To the best of our knowledge, it is the first report on the synergistic hydrolysis of lichenase by LMPOs.

To characterize the hydrolysis pattern of the synergistic action of BglsE and AST, the product profiles of the enzymatic reactions of lichenan with BglsE in the presence or absence of AST were analyzed by HPLC. The endo-hydrolysis of lichenan by BglsE produced various oligosaccharides with different degrees of polymerization (Figure 1B), and the sugar profiles obtained by the hydrolysis of AST and BglsE were similar to those obtained by the action of BglsE alone, but the addition of AST resulted in a higher overall abundance of lichenan (Figure 1C). For example, the peak area obtained by BglsE alone at 4.747 min was 278539 nC*min, while the combination of BglsE and AST was 473991 nC*min, with a synergistic effect value as high as 1.7 (Supplementary Table 1). These results also suggest that AST does not alter the endogenous hydrolysis pattern of the BglsE; instead, AST may assist BglsE to efficiently catalyze the substrate by attacking the most refractory part of the substrate, thereby generating more reducing ends to assist the BglsE in efficient catalysis of the substrate. In previous studies, similar results were observed for the synergistic effect of AST on the hydrolysis of 1,4-xylan, cellulase, and 1,3-xylan (28, 34). Where the distribution of sugars was also unaltered and indicated that AST has multiple substrate specificities.

### Effect of ratios and electron donors on AST/BglsE synergy

**FIGURE 2:**
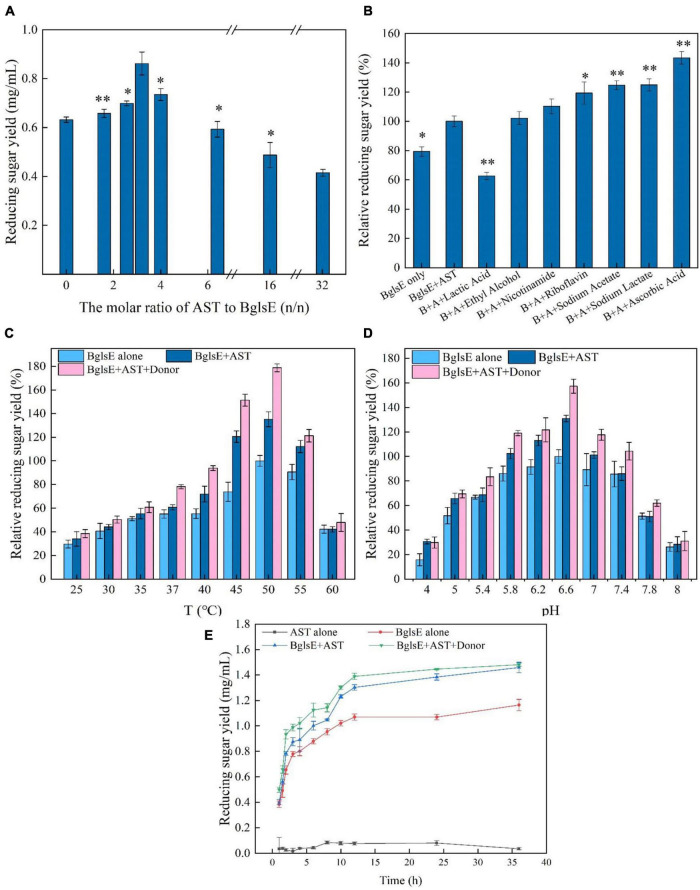
**(A)** Influences of AST/BglsE ratio on synergism in the hydrolysis of lichenin. **(B)** Influences of electron donors on synergism in the hydrolysis of lichenin. **(C)** Effect of temperature on the synergism of AST and BglsE in the hydrolysis of lichenin. **(D)** Effect of pH on the synergism of AST and BglsE in the hydrolysis of lichenin. **(E)** Time course of the synergistic effect of AST with BglsE in the hydrolysis of lichenin. *Significant difference from the control (*p* < 0.05); ^**^very significant difference from the control (*p* < 0.01).

When determining the feasibility of synergistic proteins for industrial applications, the degree of synergy, especially the amount of synergistic protein loading, is the key determinant (32, 35). Therefore, the effects of the mass ratio of AST and lichenase (0, 1.6, 2.6, 3.2, 4, 6.4, 16, to 32, BglsE was fixed at 2.4 mg/g lichenan) on synergism in lichenan hydrolysis were analyzed (Figure 2A). With the increasing ratio of AST/BglsE, the DS value firstly increased and then decreased. When the molar ratio of AST/BglsE was 3.2:1, the yield of reducing sugar reached the highest value, and the DS value was 1.31. However, a sharp downward trend was found when the ratio of AST/BglsE increased from 3.2 to 32, indicating that the synergistic effect became saturated as the increasing mole ratio, and the activity of BglsE might be inhibited by the enrichment of the intermediate products such as peroxides produced by AST (36, 37).

Lytic polysaccharide monooxygenases rely on the electron donors to activate molecular oxygen and then oxidatively cleave the glycosidic bonds of the polysaccharide chain (38, 39). Seven different types of electron donors, such as nicotinamide, lactic acid, ethanol, sodium acetate, sodium lactate, riboflavin, and ascorbic acid were loaded into the reaction system to analyze the synergistic degradation enzymatic activity (40). All donors, except lactic acid, enhanced the synergistic activity of AST/BglsE, with relative reducing sugar yields increased by 2.18 to 43.41% compared without donors (Figure 2B). When 2 mmol/L ascorbic acid was used as a reductant, the relative reducing sugar yield was increased by 43.41%. Therefore, 2 mmol/L ascorbic acid was used as the electron donor for all subsequent hydrolysis reactions.

### Effect of temperature and pH on AST/BglsE synergy

To characterize the optimum temperature of BglsE and BglsE/AST with or without 2 mmol/L ascorbic acids, the hydrolytic activities were measured at different temperatures from 25 to 60°C (Figure 2C). Interestingly, the greatest yields of reducing sugars were all observed at 50°C for both BglsE or AST/BglsE with and without electron donors, indicating that loading of AST and electron donors did not alter the optimal reaction properties of lichenase.

The optimal pH was measured by incubating BglsE and BglsE/AST with or without 2 mmol/L ascorbic acid for 2 h at pH 4.0–8.0 (Figure 2D). The optimum pH of both BglsE and BglsE/AST were 6.6, further indicating that the addition of AST and electron donor did not change the optimum reaction properties of lichenase.

### Time course of AST/BglsE synergy in lichenan hydrolysis

By incubating BglsE, AST, or BglsE/AST with lichenan for 0–36 h and comparing the yield of reducing sugars released at each enzymatic reaction (Figure 2E). AST alone did not produce detectable yields of reducing sugars from lichenan. The yield of reducing sugars produced by BglsE/AST was significantly higher at all reaction times than in the presence of BglsE alone. In particular, a 1.3-fold synergistic effect was observed at 24 h, indicating that AST did not directly hydrolyze lichenan, but increased the hydrolysis efficiency of lichenase. The substrate may need to be structurally modified by AST to improve the accessibility of the substrate with lichenase, which has difficulty in fully reaching insoluble lichenan. Furthermore, the yield of reducing sugars was 1.43 times greater than that of BglsE/AST after 2 h incubation. After 36 h, the same yield of reducing sugars was produced in the samples with and without electron donors, which may be due to those soluble small-molecule oligosaccharides produced by enzymatic degradation were already available as electron donors for AST, and the donor no longer affects the enzymatic hydrolysis process.

### Synthesis and characterization of silica nanoparticles

**FIGURE 3:**
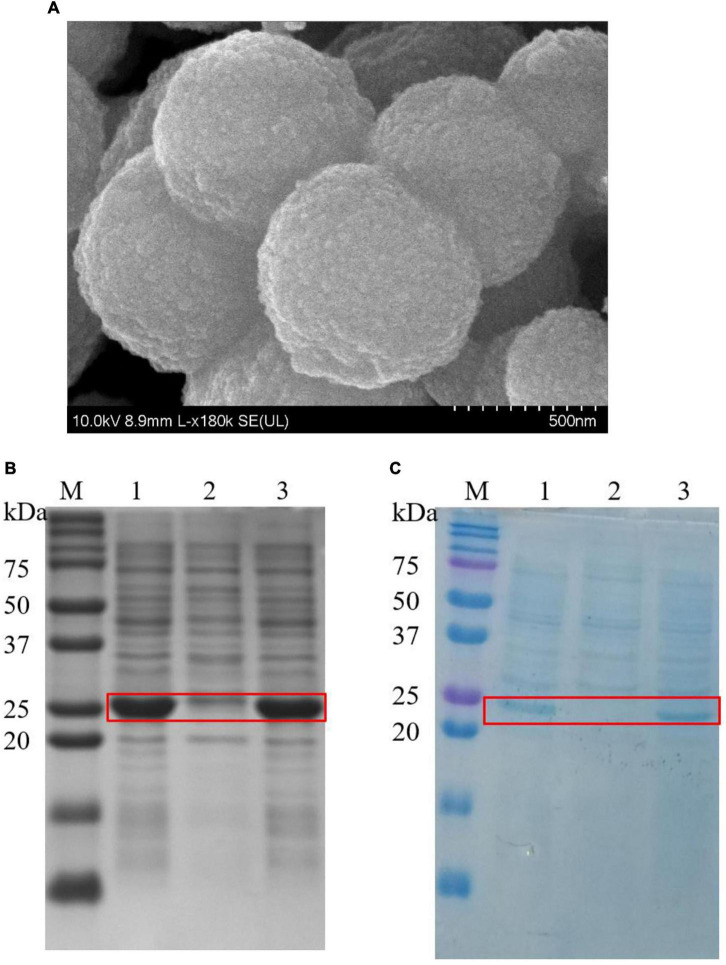
**(A)** SEM micrograph of K5-C@silica (scale bar is 0.5 μm); **(B)** The SDS-PAGE of expression, purification, and immobilization of lichenase, Red box indicates the position of the bands corresponding to BST; Lane M: marker; 1: cell lysate of BST; 2: cell lysate of BST after immobilization employing K5SC@SiO_2_; 3: cell lysate of BST after immobilization employing K5@SiO_2_; **(C)** The SDS-PAGE of expression, purification, and immobilization of AST, Red box indicates the positions of the bands corresponding to AST. 1: cell lysate of AST; 2: cell lysate of AST after immobilization employing K5SC@SiO_2_; 3: cell lysate of AST after immobilization employing K5@SiO_2_.

Cationic ELPs, which capable of biomimetic silicification was, fused with SpyCatcher to generate the bifunctional ELPs-SpyCatcher chimera (K5SC). The K5SC chimera was self-purified from cell lysates by two simple ITC cycles, and the SDS-PAGE yielded one band of 32 kDa (Supplementary Figure 2). Meanwhile, the K5SC@SiO_2_ NPs were synthesized *via* simple ELP-silicification within seconds at room temperature, which was analyzed by SEM with a diameter ranging from 500 to 800 nm (Figure 3A).

### Purification and immobilization of BST and AST in one-step

To date, most enzyme immobilization strategies need pre-purification of the target enzyme, which is a notoriously time-consuming and expensive process (21, 41). Therefore, a green and novel strategy to simultaneously purify and immobilize multi-enzymes was proposed. Briefly, 40 mg of K5SC@SiO_2_ carriers were loaded to the cell lysates containing BST and AST, respectively. After incubating at 37°C for 1 h, the silicon nanocomposites, including BST and AST *via* the SpyCather/SpyTag reaction, were collected by simple centrifugation, and the target bands of BST (28 kDa, lane 2, Figure 3B) and AST (25 kDa, lane 2, Figure 3C) in the supernatant significantly reduced, while the locations and amounts of other impurities remained unchanged. Meanwhile, the control experiment was performed with SiO_2_ carriers mediated by the ELPs without SpyCather (K5@SiO_2_). The proteins in the cell lysates of BST (lane 3, Figure 3B) and AST (lane 3, Figure 3C) did not change before and after immobilization, especially the target enzymes of BST and AST. Furthermore, the immobilized K5@silica carriers were not detected to be capable of purifying and immobilizing and have any enzyme activity. Accordingly, non-specific adsorption and immobilization of the enzyme by the silica carriers were excluded. These results demonstrate that K5SC@SiO_2_ are unique carriers that are capable of purifying and immobilizing multi-enzymes from cell lysates in one step. AST and BST have been specifically immobilized on suitably sized silica NPs carriers modified with SpyCatcher. Finally, the effectiveness of the dual-enzyme isolation and purification with integrated immobilization was characterized by four metrics. The protein loading of K5SC@SiO_2_ carriers for AST and BST were 429.54 and 487.91 μg/mg, respectively, and the immobilization yield, activity recovery, and immobilization efficiency of the captured AST/BST dual-enzyme reached 94.8, 82.9, and 87.4%, respectively. It is further proved that this is a cost-effective and environmentally friendly strategy for multi-enzyme immobilization.

### Temperature and pH on the free or immobilized dual-enzyme

**FIGURE 4:**
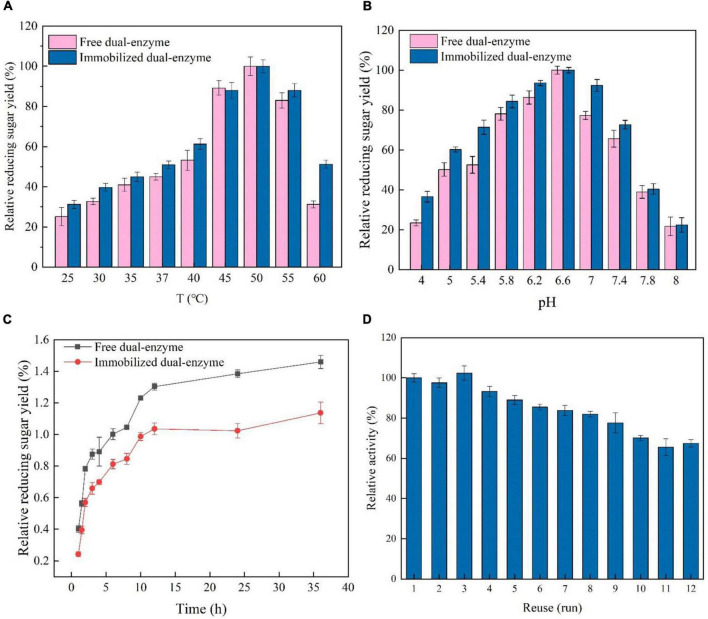
**(A)** Effect of temperature on the synergism of the immobilized and free dual-enzyme in the hydrolysis of lichenin. **(B)** Effect of pH on the synergism of the immobilized and free dual-enzyme in the hydrolysis of lichenin. **(C)** Time course of the synergism of the free or immobilized dual-enzyme in the hydrolysis of lichenin. **(D)** Reusability of the immobilized dual-enzyme.

To modulate the microenvironment of the immobilized AST/BST on K5SC@SiO_2_, the effect of temperature on the enzymatic activities was analyzed ranging from 25 to 60°C (Figure 4A). The optimum temperature of AST/BST before and after immobilization were the same, both at 50°C, indicating that the oriented and mild immobilization process would not change the optimum reaction properties of the enzymes. Furthermore, the hydrolytic activities of free and immobilized BST/AST were assayed at pH 4.0∼8.0 for 2 h (Figure 4B). Both free and immobilized BST/AST had the same influence trend of enzyme activity, and the maximum relative activities were all observed at pH 7.0, which may be due to that the dual-enzyme were immobilized on the surface of the carrier through covalent bonding, and their mild process did not significantly change the pH values of BST and AST.

### Time course of the synergistic hydrolysis by free or immobilized dual-enzyme

To investigate the effect of the immobilized procedure on the synergistic hydrolysis, the time course of the hydrolysis of lichenan by BST/AST before and after immobilization was analyzed. As shown in Figure 4C, the yield of reducing sugars for the free BST/AST was always higher than that of the immobilized form. After 36 h, the final reducing sugar yield for the free and immobilized BST/AST was 1.46 and 1.14 mg/mL, respectively. However, the reducing sugar yield of the immobilized BST/AST was 22.1% less than that of the free form. It is a normal loss by the immobilization process or by being immobilized the enzyme on the carriers, which becomes less in contact with the insoluble substrate. However, the immobilized enzyme has incomparable advantages over the free enzyme, such as relative stability and recyclability. Therefore, the immobilized enzyme is a kind of nano-biocatalysts with great potential in the bioconversion of lichen.

### Effect of metal ions on the free or immobilized dual-enzyme

The effects of 10 mmol/L metal ions and EDTA on the synergistic activity of free and immobilized AST/BST were investigated (Table 1). Ca^2+^, Ni^2+^, Ba^2+^, and Mg^2+^ promoted the synergistic activity of AST/BST before and after immobilization by 9.2–27.4%, confirming that some divalent metal ions can promote the activity of dissolved polysaccharide monooxygenase. But, Cu^2+^ showed the strongest inhibition effect. Overall, dual-enzyme are more sensitive to metal ions.

**TABLE 1:** The influences of metal ions and chemicals on the activity of the free or immobilized Bgls and AST.

Metal ion (10 mM)	RA (%) free BC	RA (%) immobilized BC	Metal ion (10 mM)	RA (%) free BC	RA (%) immobilized BC
Control	100.00	100.00			
Zn^2+^	61.58 ± 3.62	59.36 ± 1.47	Mn^2+^	59.42 ± 3.90	68.31 ± 1.08
Ca^2+^	113.12 ± 5.53	118.66 ± 0.92	Mg^2+^	113.16 ± 1.65	109.24 ± 0.87
Ni^2+^	127.21 ± 2.16	127.38 ± 0.67	K^+^	88.37 ± 2.61	97.26 ± 2.31
Co^2+^	87.48 ± 2.05	96.43 ± 3.38	Ba^2+^	117.99 ± 2.39	119.54 ± 2.47
EDTA	79.12 ± 3.68	84.31 ± 2.34	Cu^2+^	25.99 ± 3.67	41.21 ± 4.62

RA, relative activity; BC, Bgls+AST.

### Reusability of immobilized dual-enzyme

The industrial application of expensive enzymes always needs, in many instances, its reuse and recovery to make the process economical and feasible (9, 42). Covalent immobilization on the carrier is one of the most stable strategies for enzyme immobilization, which effectively reduces the chance that the enzyme falls off the carrier and thus generally achieves good reproducibility (43, 44). To analyze the reusability of immobilized AST/BST, 12 reaction cycles were performed using lichenan as substrates (Figure 4D). The immobilized AST/BST retained approximately 93.2% of the initial activity in the 4th cycle and 67.4% in the 12th cycle. Meanwhile, the immobilized BST retained approximately 91.7% of the initial activity in the 4th cycle and 66.8% in the 12th cycle, conforming that the synergistic effect of AST and immobilized AST/BST on silica NPs show excellent durability and reusability.

Adsorption, entrapment, and cross-linking are three commonly used enzyme immobilization technologies. The adsorption method has relatively weak bonding, which causes the loss of the enzyme and the detachment of the carriers during the operation; the encapsulation method is not suitable for macromolecular substrates. In contrast, cross-linking generally provides stronger bonding, less enzyme loss, and thus better reusability (45). For example, Kheirkhah reported that lipase was entrapped in the ZIF-8 carriers, retaining only 25% of original protein loading after 10 cycles (46). Cui et al. reported that the catalase adsorbed on the Fe^3+^-TA carriers retained only 20% of the initial activity (47). By drawing on these results, the cross-linked dual-enzyme on the carriers can be reused for at least 12 cycles without a big loss of initial activity, and the slight decrease in activity may be due to the loss of carriers during the process of centrifugation. In short, by combining ELP-based silicification and SpyCatcher/SpyTag-based covalent bioconjugation, we developed a novel all-in-one strategy to fabricate nanomaterials capable of target-specific covalent multi-enzyme immobilization from cell lysate without pre-purification, which have great potentials for polysaccharides bioconversion.

## Conclusion

The LPMOs named AST from *Chaetomium globosum* was verified to have 1.25-fold synergism with lichenase. HPLC results further confirmed that AST did not alter the endogenous hydrolysis mode of lichenase, but improved the hydrolysis efficiency of lichenase by breaking the long chain at the reducing end of polysaccharides. To the best of our knowledge, it is the first report of the synergistic effect of LPMOs with lichenase, which may have great synergistic potential in the hydrolysis of lichen biomass. Meanwhile, a green and novel strategy for the covalent immobilization of dual-enzyme (LMPOs and lichenase) on SNPs directly from the cell lysate was proposed. The immobilized dual-enzyme showed excellent immobilization efficiency (87.4%) and good reusability. Further confirms the practicality of the immobilization strategy, which has obvious advantages in the bioconversion of lichen biomass.

## Data availability statement

The original contributions presented in this study are included in the article/Supplementary material, further inquiries can be directed to the corresponding author.

## Author contributions

LC designed and performed the experiment and wrote the original draft. YZ performed enzyme experiments. YC did statistic analysis. YL performed enzyme characterization. LL investigated and validated the experiments. GZ reviewed and edited the manuscript. All authors read and approved the submitted manuscript.
