# Supplementary material for: The synergism of lytic polysaccharide monooxygenases with lichenase and their co-immobilization on silica nanospheres for green conversion of lichen biomass
Source: Front Nutr. 2022 Oct 19;9:970540. doi: 10.3389/fnut.2022.970540 (PMC9626761; doi:10.3389/fnut.2022.970540)
Supplement: Supplementary file 1 [file Data_Sheet_1.docx]

Supplementary Materials

**The synergism of lytic polysaccharide monooxygenases with lichenase and their co-immobilization on silica nanospheres for green conversion of lichen biomass**

Lixi Cai^1, 2，3^, Ying Zheng^4^, Yunmeng Chu^2^, Yuanqing Lin^2^, Lixing Liu^1, 3^, Guangya Zhang^2⁎^


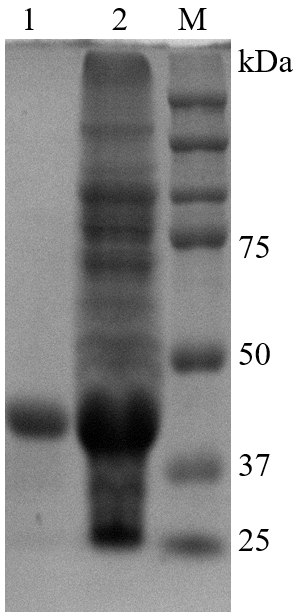

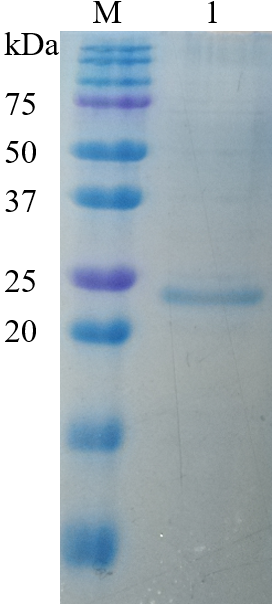


**A**

**B**

**Figure S1** The SDS-PAGE of the purified protein; **(A)** The recombinant lichenase. M: protein molecular weight marker; lane 1: BglsE; lane 2: the cell lysate of BglsE; **(B)** The recombinant LPMOs. lane 1: the SDS-PAGE of AST.

**
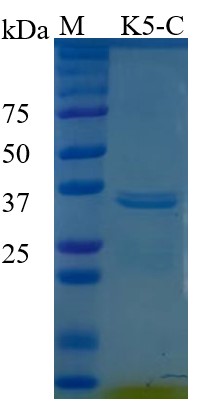
**

**Figure S2** The SDS-PAGE of K5SC;

TABLE S1 | **The hydrolysis products from lichenin were analyzed by HPLC**

| **Time(min)** | **Peak area of lichenase** | **Time(min)** | **Peak area of lichenase+AST** | **DS** |
| --- | --- | --- | --- | --- |
| 4.384 | 6.9887*10^4^ | 4.337 | 5.42642*10^4^ | 0.7765 |
| 4.767 | 2.78539*10^5^ | 4.776 | 4.73991*10^5^ | 1.7017 |
| 5.997 | 4.324*10^5^ | 5.969 | 5.04065*10^5^ | 1.1657 |
| 7.614 | 1.2446*10^5^ | 7.664 | 1.61329*10^5^ | 1.2962 |
| 10.767 | 3.40829*10^4^ | 11.112 | 3.02804*10^4^ | 0.8884 |
| 13.656 | 1.81239*10^4^ | 13.563 | 2.42625*10^4^ | 1.3387 |
| 14.884 | 1.0648**10^4^ | 14.885 | 6984.61 | 0.6559 |
| 18.688 | 788.304 | 18.612 | 6051.661 | 7.6789 |
| Total: | 9.57243*10^5^ |  | 1.26306*10^6^ | 1.3196 |

DS was the degree of synergy by AST
